# Human Activity Recognition in a Free-Living Environment Using an Ear-Worn Motion Sensor

**DOI:** 10.3390/s24092665

**Published:** 2024-04-23

**Authors:** Lukas Boborzi, Julian Decker, Razieh Rezaei, Roman Schniepp, Max Wuehr

**Affiliations:** 1 German Center for Vertigo and Balance Disorders (DSGZ), Ludwig-Maximilians-University of Munich, 81377 Munich, Germany; 2 Institute for Emergency Medicine and Medical Management, Ludwig-Maximilians-University of Munich, 80336 Munich, Germany; 3 Department of Neurology, Ludwig-Maximilians-University of Munich, 81377 Munich, Germany

**Keywords:** human activity recognition, inertial sensor, ear, in-ear sensing, vital sign monitoring, wearables, machine learning, deep learning

## Abstract

Human activity recognition (HAR) technology enables continuous behavior monitoring, which is particularly valuable in healthcare. This study investigates the viability of using an ear-worn motion sensor for classifying daily activities, including lying, sitting/standing, walking, ascending stairs, descending stairs, and running. Fifty healthy participants (between 20 and 47 years old) engaged in these activities while under monitoring. Various machine learning algorithms, ranging from interpretable shallow models to state-of-the-art deep learning approaches designed for HAR (i.e., *DeepConvLSTM* and *ConvTransformer*), were employed for classification. The results demonstrate the ear sensor’s efficacy, with deep learning models achieving a 98% accuracy rate of classification. The obtained classification models are agnostic regarding which ear the sensor is worn and robust against moderate variations in sensor orientation (e.g., due to differences in auricle anatomy), meaning no initial calibration of the sensor orientation is required. The study underscores the ear’s efficacy as a suitable site for monitoring human daily activity and suggests its potential for combining HAR with in-ear vital sign monitoring. This approach offers a practical method for comprehensive health monitoring by integrating sensors in a single anatomical location. This integration facilitates individualized health assessments, with potential applications in tele-monitoring, personalized health insights, and optimizing athletic training regimes.

## 1. Introduction

Human activity recognition (HAR) has emerged as a transformative technology enabling the continuous monitoring of both static and dynamic behaviors in diverse environmental conditions [1]. Its applications extend across industries, sports, and healthcare [2]. With respect to the healthcare sector, continuous HAR monitoring presents an opportunity to obtain ecologically valid information about a patient’s condition in everyday environments and facilitates the registration of rare and fluctuating events that are often missed in stationary short-term clinical assessments [3]. This technology holds significant potential for identifying diseases in their pre-clinical stages, monitoring disease progression or evaluating the effects of interventions, and detecting critical events such as falls [4,5,6,7,8,9].

The technical landscape of HAR in healthcare applications predominantly relies on wearable motion sensors, primarily accelerometers, often augmented with gyroscopes or magnetometers. The small size and low energy consumption of these sensors allow them to be placed either directly on different body parts or to be integrated into wearable devices (e.g., smartwatches). The placement location on the body is strategically chosen so that the motion profile captured by the sensor can identify and distinguish various forms of activities as effectively as possible. Common choices for mounting include the lower back [10], thighs [8,11], feet (often integrated into shoes) [12], and wrist (often integrated into smartwatches or fitness trackers) [13], balancing unobtrusiveness and seamless integration into daily life.

In this study, we explore an alternative approach by utilizing an ear-worn sensor for HAR [14,15]. The ear, in contrast to the lower extremities or the trunk, has several unique advantages for connecting an HAR device. The head, housing crucial sensory peripheries for vision, audition, and balance, remains exceptionally stable during various movements [16,17], providing a reliable locus for low-noise identification and differentiation of different bodily activities. Additionally, the ear is a location where users in particular in the elderly population often already employ assistive devices like hearing aids or eyeglass frames that could be readily combined with a miniature motion sensor [18]. Finally, beyond motion, the ear is an ideal location for monitoring a person’s physical and health status, as optical in-ear sensors can reliably capture vital signs such as heart rate, blood pressure, body temperature, and oxygen saturation [19]. Therefore, the ear stands out as a promising candidate site for wearing a single integrative sensing device, facilitating comprehensive continuous monitoring of a person’s activity and health status in everyday life.

The primary objective of this study was to investigate the potential of an ear-worn motion sensor, integrated into an in-ear vital sign monitor, to classify various human activities. In a large group of healthy individuals, daily activities such as lying, sitting, standing, walking, running, and stair walking were recorded and labeled in a free-living environment. To achieve a classification of these activities, we employed machine learning algorithms, adopting a two-fold strategy: shallow machine learning models utilizing interpretable features or parametrizations of movement (e.g., movement amplitude and variance) and state-of-the-art deep learning algorithms specifically designed for HAR. This dual-pronged approach aims to explore the interpretability of features and the robust classification capabilities offered by deep learning in the context of inertial-sensor-based HAR. Through these contributions, we aim to provide insights into the potential of ear-worn sensors for enhancing healthcare monitoring and disease management.

## 2. Materials and Methods

### 2.1. Participants

Fifty healthy individuals, between 20 and 47 years old (age: 29.4 ± 6.5 years; height: 1.73 ± 0.10 m; weight: 70.8 ± 15.8 kg; 25 females), participated in the study. All participants signed written informed consent prior to inclusion and were screened for any neurological or orthopedic conditions that would influence either balance or locomotion.

### 2.2. Ear-Worn Motion Sensor

**Figure 1:**
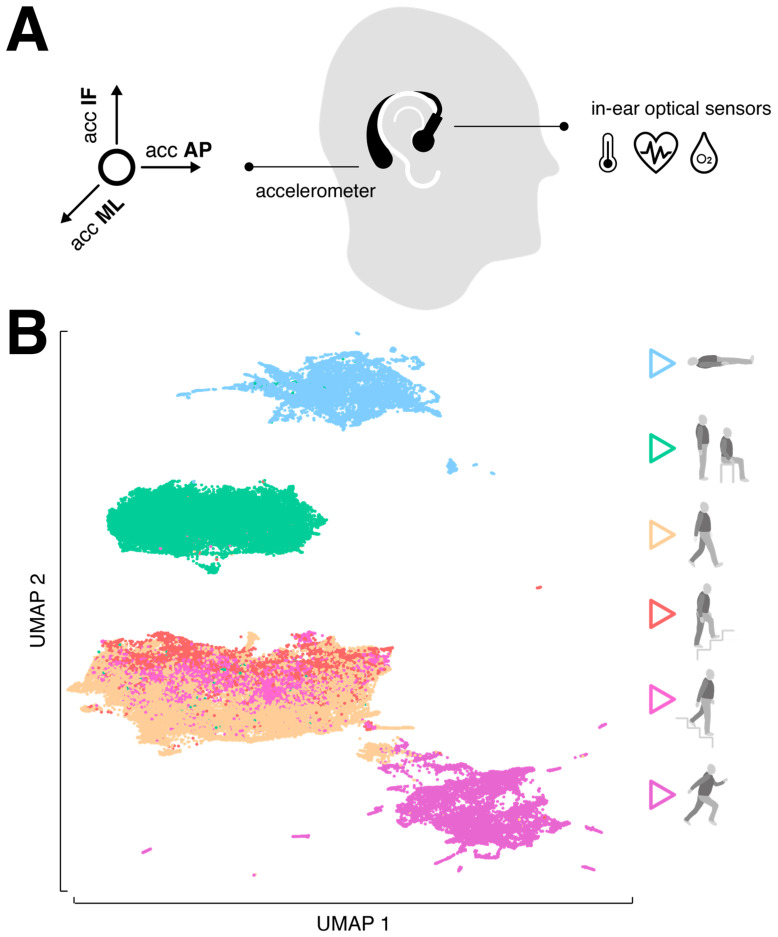
Sensor specification and distribution of activity classes. (**A**) The in-ear wearable vital sign monitor: optical sensors are located on the sensor earplug that goes into the outer ear canal, while the triaxial accelerometer is located in the processing unit behind the auricle. (**B**) Low-dimensional embedding of the 6 activity classes (lying, standing/sitting, walking, ascending stairs, descending stairs, running) via UMAP. Abbreviations: AP: anterior–posterior axis; ML: medio-lateral axis, IF: inferior–superior axis; UMAP: Uniform Manifold Approximation and Projection.

The motion sensor consisted of a triaxial accelerometer (range: ±16 g; accuracy: 0.0002 g; sampling rate: 100 Hz), which is integrated into a commercial, wearable in-ear vital sign monitor (c-med° alpha, size: 55.2 mm × 58.6 mm × 10.0 mm; weight: 7 g, Cosinuss GmbH, Munich, Germany). The vital sign monitor consists of a silicon earplug that is in contact with the outer ear canal skin and contains an infrared thermometer for recording body temperature and an optical sensor for measuring pulse rate and blood oxygen saturation. The earplug is connected to an earpiece hooked around the ear conch, in which the motion sensor is located (Figure 1A). The wearable device transmits acquired motion and vital signals in real time via Bluetooth Low Energy to a gateway that subsequently streams this information into the cosinuss° Health server. The server platform can be accessed via a smartphone application to monitor the acquired signals in real time and to add real-time annotations (activity labels) to the recording (see ground truth annotation in Section 2.3 Experimental procedures).

### 2.3. Experimental Procedures

The experiments were conducted in the university research building and outdoors (urban environment). Initially, each participant was briefed on the experimental procedures. Subsequently, two ear-worn sensors were attached to the left and right ears with the aim of training and obtaining an algorithm for activity classification that works independently of the attachment side.

The focus of activity classification was on so-called low-level activities, characterized by a sequence of body movements and postures, typically lasting a few seconds to several minutes [20]. The recorded activity forms included lying, sitting, standing, walking, ascending or descending stairs, and running (Figure 1B). To make the activity classification robust for everyday variations, participants were encouraged to perform them as naturally as possible. For example, lying also included turning or restlessly lying in bed, standing involved tapping in place or chatting, and walking was performed at varying slow, comfortable or fast speeds. The activities lying and stair climbing were exclusively recorded indoors, while the other activities were recorded both indoors and outdoors. Each participant performed all activities multiple times in a pseudorandomized order. The average recording duration was approximately 30 min per participant.

An experimenter accompanied the participant throughout the entire experiment and instructed them on when to end one activity and start a new one. The experimenter simultaneously performed ground truth annotation (activity type) by annotating the real-time sensor time series with the respective activity label via a smartphone application. The activity label was assigned for the period shortly after the onset of the activity until its termination. Transitions between activities or brief interruptions in the experimental procedure did not receive a label.

In preliminary analyses, it was observed that the activity classes of sitting and standing were fundamentally indistinguishable, which is expected, as the head behaves in the same orientation during both activities. Therefore, the two classes were combined for further investigations resulting in a total of six activity classes: lying, sitting/standing, walking, ascending stairs, descending stairs, and running.

### 2.4. Classification Models

#### 2.4.1. Data Segmentation

The acquired three-dimensional motion time series (anterior–posterior dimension: AP; superior–inferior dimension: SI; medio-lateral dimension: ML) were segmented by using the sliding window technique employing various sizes of non-overlapping windows (i.e., 0.5, 1, 2 s) to determine an optimal configuration. These sizes correspond to the commonly used window sizes in HAR applications, reflecting the average duration of basic everyday activities [21,22]. Only recording sequences with an activity label were used, and simultaneously, it was ensured that each recording segment contained only a unique activity label.

#### 2.4.2. Shallow Learning Models

After segmentation, a set of statistical features established for time series analysis of physiological signals and previously applied in HAR were computed per segment [23,24,25,26]. These included the mean, mean of sum of absolute values, minimum, maximum, range, sum, standard deviation, variance, root-mean-square, interquartile range, zero-crossing rate, skewness, kurtosis, signal energy, and spectral entropy that were computed per motion axis as well as for the acceleration magnitude vector (${acc}_{AP}$, ${acc}_{SI}$, ${acc}_{ML}$, ${acc}_{magn}$). In addition, the Pearson and Kendall correlation coefficients were computed for every combination between motion axes, resulting in a total number of 64 features.

For pre-evaluation, a set of eight standard machine learning models were trained, including *K-Nearest Neighbors*, *Decision Tree*, *Support Vector Machine*, *Naive Bayes*, *Bagging*, *Random Forest*, *ExtraTrees*, and *Gradient Boosting*. Input features were first normalized by applying a transformation to zero-mean and unit-variance distribution, and subsequently, each model was trained using a stratified 10-fold cross-validation that ensured that data from one participant were only represented in either the training or testing set. From the set of all model and sliding window combinations tested, the model with the highest accuracy was selected for further hyperparameter optimization using grid search with cross-validation. Finally, feature selection on the optimized classifier was performed using univariate statistical comparisons (i.e., ANOVA F-value between features) to identify a parsimonious set of the most informative features that still ensure high classification accuracy.

#### 2.4.3. Deep Learning Models

Besides shallow learning models, which use pre-engineered motion features, the performance of deep learning models that automate feature extraction from raw sensor inputs was evaluated. Two deep learning models specifically designed for the task of HAR on wearable motion signals were considered. The *DeepConvLSTM* architecture combines a convolutional neural network with a long short-term memory recurrent network (LSTM) and has been widely applied in the past [27]. The model employs a series of convolutional layers that learn to extract essential features from the raw motion time series followed by LSTM layers that model their temporal dependencies. The *ConvTransformer* based on a combination of a convolutional neural network with a transformer model is a more recent model that achieves state-of-the-art performance on many publicly available datasets [28]. The model initially utilizes a convolutional layer to model the local information of the motion time series and then uses a transformer to represent the temporal information of the modeled signal features and adds an attention mechanism to determine the essential features.

The two deep learning models were trained for the same window sizes as described above. Initially, raw motion sensor data were normalized by applying a transformation to zero-mean and unit-variance distribution, and subsequently, each model was trained using a stratified 10-fold cross-validation that ensured that data from one participant were only represented in either the training or testing set. Both models are trained to reduce categorical cross-entropy loss using the Adam optimizer.

#### 2.4.4. Performance Metrics and Implementation

The performance of the different studied shallow and deep learning models was evaluated based on the number of correctly recognized activities (true positives; TPs), the number of incorrectly recognized activities (false positives; FPs), the number of correctly rejected activities (true negative; TNs), and the number of incorrectly rejected activities (false negative; FNs). Based on these numbers, model performance war primarily evaluated by the weighted *F1-Score*, which considers both precision and recall while considering imbalances in class distribution. It calculates the harmonic mean of precision and recall and ranges between 1 and 0 reflecting the best and worst performance, respectively:

$$precision=\frac{TP}{TP+FP},$$

$$recall=\frac{TP}{TP+FN},$$

$$F1=\frac{2*precision*recall}{precision+recall},$$

All analyses and models were implemented in Python 3.9 using scikit-learn 1.3 and the Keras API 2.10 with TensorFlow backend.

## 3. Results

### 3.1. Dataset Characteristics

A total of 50.8 h (left ear-worn sensors: 27.0 h; right ear-worn sensors: 23.8 h) of activity was recorded from the 50 participants. In six participants, sensor data were only available from one ear-worn device due to transmission or battery issues occurring during recording. The distribution of recorded activity classes and the corresponding duration statistics for single periods of activity are shown in Table 1. Recorded activities had a varying duration between 3 and 200 s. The final dataset reveals a certain imbalance between the measured activity classes, with walking dominating at 31% of all cases, and descending stairs being the least represented at only 6% of the cases. To address this imbalance between activity classes in the subsequent analyses, each classification model was trained using a stratified cross-validation, and the model performance was evaluated based on the weighted F1-score, which considers imbalances between the classes.

### 3.2. Shallow Learning Models

Table 2 shows the results of the pre-evaluation for various window sizes used for data segmentation and different shallow machine learning models. All models achieved better classification rates for longer window sizes. Across all examined window sizes, *Support Vector Machine* was leading in classification, achieving the best accuracy of 93% with a window size of 2 s.

Hyperparameter optimization of this classifier performed using grid search with cross-validation yielded a gamma value of 0.0001 and a regularization parameter of C of 10,000. Table 3 presents the classification report of this optimized model with an average classification accuracy of 95%. It almost perfectly identified and differentiated between the activities lying, sitting/standing, and running but still showed some difficulties in distinguishing between the activities walking and ascending and descending stairs.

In a final step, a feature selection was performed to obtain a parsimonious model with a concise number of parameters without significantly compromising classification accuracy. This procedure revealed that a model based on ten signal features was sufficient to yield a classification accuracy above 91% (Table 4). The top-ranked features reflected basic time-domain characteristics (e.g., variance, range, minimum) of the up and down head acceleration and the acceleration magnitude vector (${acc}_{SI}$ and ${acc}_{magn}$).

### 3.3. Deep Learning Models

Both examined deep learning models showed an overall improved classification compared to the optimized best-performing shallow learning model. The increase in classification accuracy ranged between 2 and 3%. The more recent model, *ConvTransformer*, did not show any advantage over the *DeepConvLSTM* network. Table 5 displays the classification performance of both networks for various window sizes. 

**Figure 2:**
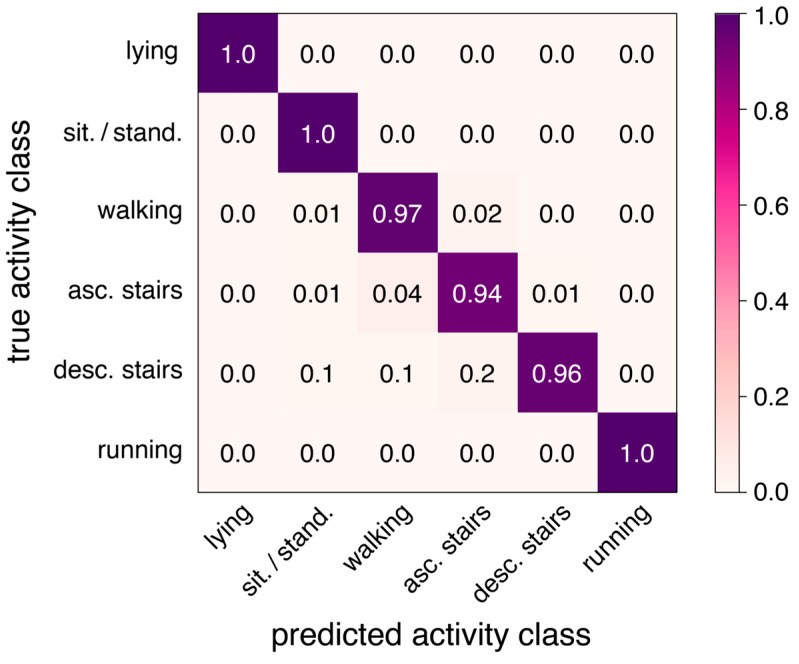
Confusion matrix of the best-performing model (*DeepConvLSTM* with a window size of 2s) yielding an overall accuracy of 98% for the 6 activity classes.

Table 6 presents the classification report for the best configuration yielding an average classification accuracy of 98% (*DeepConvLSTM* with a window size of 2 s), and Figure 2 presents the corresponding confusion matrix.

## 4. Discussion

The primary objective of this study was to assess the efficacy of an ear-mounted motion sensor in classifying various human activities. Our findings substantiate the ear’s suitability as a measurement site for an activity monitor, given its stability during diverse movements and the potential integration with commonly worn assistive devices, such as hearing aids and eyeglass frames. Moreover, the ear’s capacity to host both motion and vital sign sensors positions it as a promising location for comprehensive health monitoring. The application of state-of-the-art deep learning models yielded excellent results, achieving a 98% accuracy in classifying six common activities, which surpasses previous equivalent approaches [14,15] and is comparable to current benchmark activity classifiers using one or multiple motion sensors at the trunk or lower extremities. Furthermore, even a conventional shallow network demonstrated compelling performance using a concise set of interpretable statistical features. It should be emphasized that the yielded classification algorithm is agnostic regarding which ear the sensor is worn and robust against moderate variations in sensor orientation (e.g., due to differences in auricle anatomy), meaning no initial calibration of the sensor orientation is required.

The study incorporated a diverse cohort of participants, spanning various ages, genders, and body dimensions, engaging in activities in natural, urban environments. While the algorithm successfully classified a wide range of activities, a limitation emerged in differentiating between sitting and standing, a challenge also observed in previous trunk-mounted approaches [29]. Future enhancements could involve considering postural transitions, potentially allowing for the distinction between these two activities. Additionally, the current algorithm did not account for active (e.g., biking) or passive (e.g., riding a car, subway) transportation, suggesting room for expansion to create a more comprehensive activity monitor. Finally, further studies are necessary to assess the efficacy of our activity recognition algorithm in older individuals or clinical populations (e.g., patients with musculoskeletal or neurological gait disorders), whose everyday movement patterns may diverge considerably from those of the healthy population focused on in this study.

**Figure 3:**
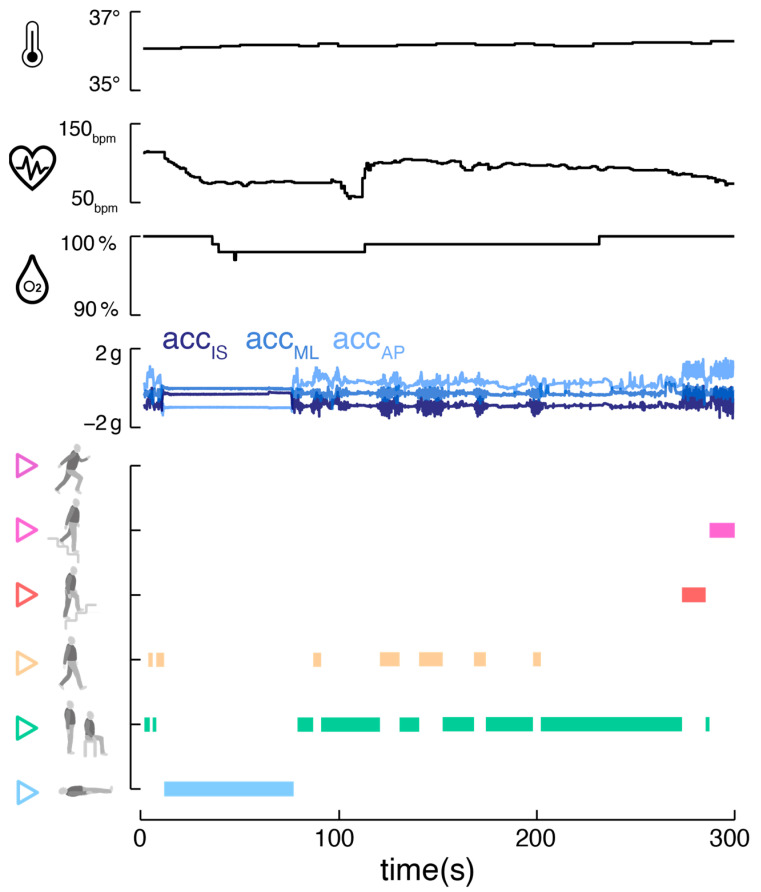
Combined activity and vital sign monitoring. Exemplary activity classifier output (*DeepConvLSTM* with window size of 2 s) alongside corresponding vital sign parameters (body temperature, pulse rate, oxygen saturation) derived from a single ear-worn wearable.

The integration of an ear-worn motion sensor with a vital sign monitor offers promising advantages (Figure 3). The parallel monitoring of bodily activity and vital signs, including temperature, pulse rate, and oxygen saturation, allows a comprehensive view of an individual’s health status. This approach is particularly beneficial in tele-monitoring applications, where understanding the context and behavior is crucial for accurate feedback [30,31,32]. Correlating vital signs with specific activities aids in establishing individual baselines and identifying anomalies that may indicate health issues [33,34,35]. Long-term analysis of these correlations could provide personalized health insights, identifying individual behavioral patterns and habits that might impact health. For athletes, understanding how vital signs respond to different exercise intensities can help to optimize training regimes and prevent overtraining [36]. The motion sensor could finally contribute to improving the accuracy of vital sign readings by automatically detecting and addressing motion artifacts through algorithm tuning [19].

## 5. Conclusions

This study demonstrates the effectiveness of an ear-worn motion sensor for highly accurate classification of common sedative and ambulatory activities. When combined with an in-ear vital sign monitor, this integrated system could enhance the accuracy of vital sign readings and enable the study of correlations between vital signs and activities. Follow-up studies are required to evaluate the reliability of the activity monitor in non-healthy clinical populations. Future applications should further expand the activity monitor to include activities like active and passive transportation, and efforts should be directed towards training algorithms for more detailed activity classification, such as characterizing step-to-step patterns during ambulatory activities [18]. This research lays the foundation for a comprehensive and versatile ear-centered monitoring system with potential applications in healthcare, sports, and beyond.

## Figures and Tables

**Table 1:** Distribution of recorded activities and corresponding duration statistics of single activity periods.

Activity	Percentage	Mean (s)	Std (s)	Min (s)	Max (s)
lying	12.8	62.0	10.5	30.0	100.4
sitting/standing	29.0	25.8	14.1	3.0	78.2
walking	30.6	66.7	28.2	12	199.1
ascending stairs	7.1	8.6	1.1	4.9	12.4
descending stairs	6.3	7.7	1.8	4.3	33.2
running	14.2	32.4	9.5	9.9	84.4

**Table 2:** Pre-evaluation of different shallow learning models on varying window sizes used for data segmentation. The best configuration (classifier and window size) is underlined.

Classifier	Win Size	0.5 s	1 s	2 s
*K-Nearest Neighbors*		0.890	0.892	0.889
*Decision Tree*		0.868	0.882	0.879
*Support Vector Machine*		0.924	0.929	0.930
*Naive Bayes*		0.861	0.867	0.870
*Bagging*		0.903	0.910	0.910
*Random Forest*		0.911	0.918	0.917
*ExtraTrees*		0.906	0.911	0.911
*Gradient Boosting*		0.921	0.925	0.925

**Table 3:** Classification report of the optimized (grid search cross-validation) best-performing configuration, i.e., *Support Vector Machine* with a window size of 2 s.

Activity	Precision	Recall	F1-Score	Support
lying	0.987	0.997	0.992	8095
sitting/standing	0.984	0.997	0.990	16,410
walking	0.939	0.936	0.937	19,288
ascending stairs	0.700	0.690	0.695	2549
descending stairs	0.802	0.828	0.815	2001
running	0.995	0.965	0.979	8367
accuracy			0.951	56,710
macro avg	0.901	0.902	0.901	56,710
weighted avg	0.951	0.951	0.951	56,710

**Table 4:** Outcomes from feature selection performed on the optimized best-performing configuration.

(1) min ${acc}_{SI}$	(2) min ${acc}_{magn}$	(3) min ${acc}_{magn}$
(5) std ${acc}_{SI}$	(6) std ${acc}_{magn}$	(7) iqr ${acc}_{SI}$
(9) range ${acc}_{SI}$	(10) range ${acc}_{magn}$	

min: minimum; var: variance; std: standard deviation; iqr: interquartile range.

**Table 5:** Pre-evaluation of different deep learning models on varying window sizes used for data segmentation. The best configuration (classifier and window size) is underlined.

Classifier	Win Size	0.5 s	1 s	2 s
*DeepConvLSTM*		0.964	0.975	0.981
*ConvTransformer*		0.966	0.978	0.977

**Table 6:** Classification report of the best-performing model, i.e., *DeepConvLSTM* with a window size of 2 s.

Activity	Precision	Recall	F1-Score	Support
lying	0.985	0.999	0.992	6010
sitting/standing	0.986	0.997	0.992	13,518
walking	0.987	0.967	0.977	14,262
ascending stairs	0.907	0.937	0.922	3285
descending stairs	0.970	0.959	0.965	2940
running	0.998	0.996	0.997	6624
accuracy			0.981	46,639
macro avg	0.972	0.976	0.974	46,639
weighted avg	0.981	0.981	0.981	46,639

## Data Availability

The datasets used and/or analyzed during the current study will be available from the corresponding author upon reasonable request.
